# Towards identification of protein–protein interaction stabilizers *via* inhibitory peptide-fragment hybrids using templated fragment ligation†

**DOI:** 10.1039/d2cb00025c

**Published:** 2022-04-01

**Authors:** Sonja Srdanović, Zsofia Hegedüs, Stuart L. Warriner, Andrew J. Wilson

**Affiliations:** Astbury Centre for Structural Molecular Biology, University of Leeds, Woodhouse Lane Leeds LS2 9JT UK a.j.wilson@leeds.ac.uk; School of Chemistry, University of Leeds, Woodhouse Lane Leeds LS2 9JT UK; Department of Medical Chemistry, University of Szeged Dóm tér 8 H-6720 Szeged Hungary

## Abstract

Using the *h*DMX/14-3-3 interaction, acylhydrazone-based ligand-directed fragment ligation was used to identify protein–protein interaction (PPI) inhibitory peptide-fragment hybrids. Separation of the peptide-fragment hybrids into the components yielded fragments that stabilized the *h*DMX/14-3-3 interaction.

## Abstract 2

Ligand-directed dynamic-ligation is used to identify fragments that can augment the inhibitory potency of a peptide ligand and simultaneously serve as starting points for future development of PPI stabilizers.

## Graphical abstract

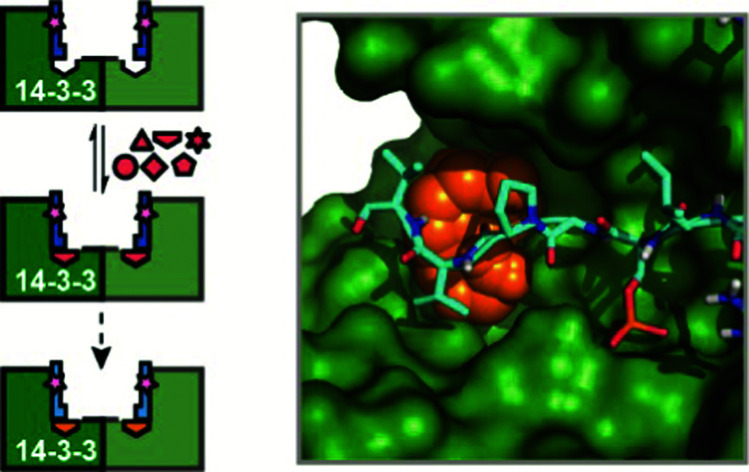

Despite continued development of new ligand discovery methods,^1,2^ it remains challenging to identify hits for a significant palette of targets *e.g.* protein–protein interaction (PPI) inhibitors/stabilizers.^3–6^ Fragment-based-drug-discovery (FBDD)^7,8^ has proven powerful in furnishing starting points for PPI inhibitor candidates and led to development of clinically approved drugs.^9^ However, identification of fragments that can serve as starting points for the elaboration of PPI stabilizers^10^ presents specific challenges given the need to form a termolecular complex paired with typically weak fragment binding. Disulfide tethering^11–14^ has proven useful in developing PPI inhibitors,^15^ allosteric modulators,^16^ and tools to study aggregation.^17^ Recently the method was shown to be useful for site-directed fragment identification using the 14-3-3*σ*/ERα interface as a model. 40-Fold stabilization was achieved for binding of a C-terminal ERα peptide by 14-3-3 in the presence of the protein-fragment adduct,^18^ although stabilization in the absence of the disulfide tether remains to be reported. The approach has been inverted to exploit cysteines present within a peptide ligand to create “covalent-molecular glues”; fragment-peptide hybrids linked *via* a disulfide.^19^ Similarly, imine based tethering has subsequently been described whereby aldehydes react with surface exposed lysine residues on the surface of 14-3-3 to stabilize its interaction with peptides.^20,21^ A further complementary approach, whereby a scaffold compatible with dynamic combinatorial exchange by virtue of a central acylhydrazone bond, was used as a template to identify small-molecule 14-3-3*ζ*/synaptopodin stabilizers.^22^

We recently used ligand-directed fragment ligation as a tool to identify peptide-fragment hybrids that inhibit a β-strand mediated PPI.^23^ Reliant on hydrazone exchange,^24,25^ this approach is advantageous in that it unites the synthesis and assay steps within a single step and rapidly explores SAR using commercially available aldehyde fragments. Inspired also by the work of Ohkanda on the use of oxime ligation for *in situ* assembly of a bivalent diterpene-peptide conjugate as an intracellular 14-3-3*ζ* inhibitor,^26^ in this work we used our recently reported data on the *h*DMX/14-3-3 interaction^27^ as a model to further elaborate acylhydrazone-based ligand-directed fragment ligation to identify PPI inhibitory peptide-fragment hybrids which upon separation into the components can yield fragments as candidate PPI stabilizers (Fig. 1a). In contrast to the earlier work on covalent protein-^18^ and peptide-based^19^ molecular glues, this approach allowed us to reveal that non-covalent fragments which stabilize PPIs can be identified using site-directed ligation methods. Furthermore, the two methods are complementary in terms of the conditions under which the bonds are stable and under which exchange occurs.^28^ Finally, the asymmetry of the hydrazone linkage when compared to the disulfide, may be advantageous in terms of yielding only unsymmetrical peptide fragment hybrids.

**Fig. 1:**
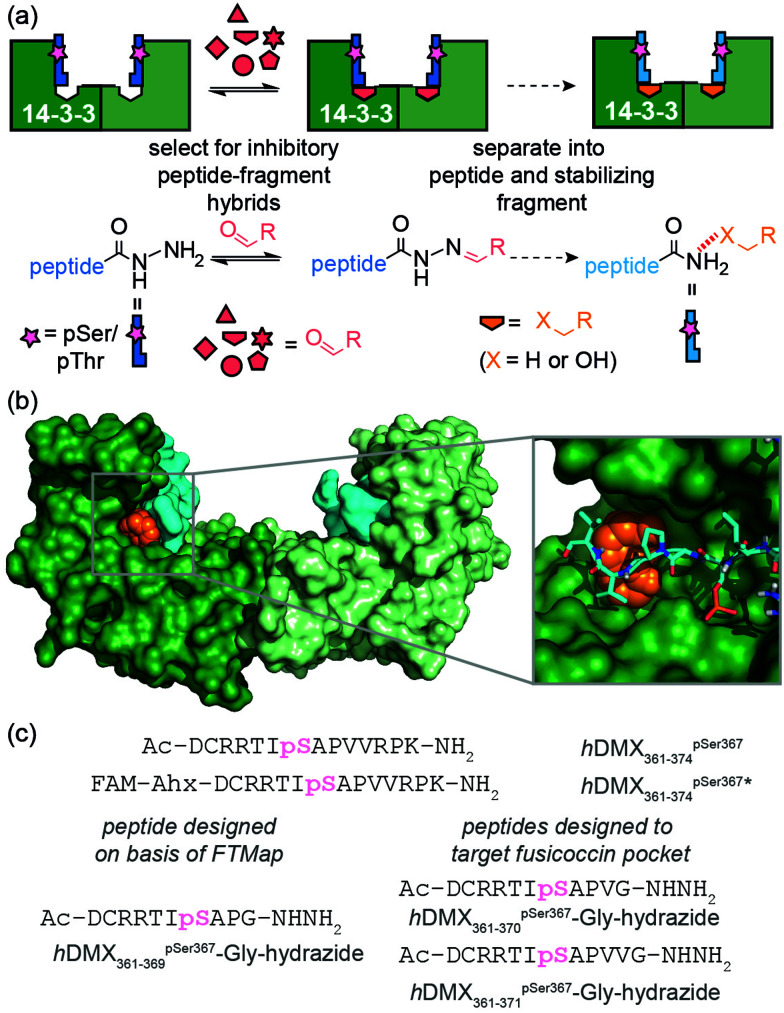
Design of acylhydrazone peptides for identification of PPI inhibitory fragment-peptide hybrids and stabilizing fragments; (a) schematic representation of dynamic ligand-directed fragment ligation approach. Reversible acylhydrazone reactions are used to generate peptide fragment hybrids using a template protein (here: 14-3-3*η*); inhibitors are then separated into peptide and fragment components for evaluation as stabilizers; (b) a cluster of small organic probes (orange spheres) were modelled at the interface between *h*DMX_361–374_^pSer367^ (cyan sticks) and 14-3-3*σ*-Δ*C* dimer (green) using FTMap (c) peptide sequences used.

The central binding groove of 14-3-3 proteins accommodates phosphorylated peptides. Beside a phospho-binding groove on 14-3-3 proteins, a defined pocket has been shown to recognize Fusicoccin and other ligands resulting in stabilization of certain client/14-3-3 interactions.^29^*h*DMX_361–374_^pSer367^ was found to partially occupy the Fusicoccin binding pocket in the co-crystal structure with 14-3-3*σ*-Δ*C*. Thus, the challenge was to identify novel ligandable sites^30^ between 14-3-3 proteins and *h*DMX_361–374_^pSer367^. Computational ‘solvent mapping’ using FTMap^31^ was therefore used to identify suitable targetable pockets on the *h*DMX_361–374_^pSer367^/14-3-3*σ* interface. For the *h*DMX_361–374_^pSer367^/14-3-3*σ*-Δ*C* structure, FTMap identified several consensus sites on the 14-3-3 protein dimer interface but only one high quality consensus site was found to occupy a pocket on 14-3-3 abutting *h*DMX_361–374_^pSer367^ (Fig. 1b and Fig. S1, ESI†), To probe this pocket using a peptide based acylhydrazide, a truncated version of *h*DMX_361–374_^pSer367^; *h*DMX_361–369_^pSer367^Gly-hydrazide (Fig. 1c) was synthesized using hydrazone resin.^32^ The key residues (Arg at position −3 and Pro at position +2) needed to maximise binding affinity were kept, whilst a C-terminal Gly was used to maximize resin loading and conformational flexibility proximal to the acylhydrazide to support exploration of the 14-3-3 surface.

A fluorescence anisotropy (FA) competition assay was subsequently carried out using conditions previously found suitable for acylhydrazone formation (pH 6.5, 50 mM NH_4_OAc, 10 mM aniline) with added DTT (1 mM) to supress disulfide formation. *h*DMX_361–369_^pSer367^Gly-hydrazide was used to compete with a tracer peptide: *h*DMX_361–374_^pSer367^* for binding to 14-3-3*η* (Fig S2, ESI†). The resulting IC_50_ values were comparable over 24 hrs indicating good stability over the expected timeframe for hydrazone exchange. Initial attempts to screen in cocktails, established the aldehydes had no affect on their own (Fig. S3, ESI†). We then performed an assay where each aldehyde was screened individually with *h*DMX_361–369_^pSer367^Gly-hydrazide. A decrease in anisotropy can be attributed to a particular hydrazone product, avoiding the additional step of product identification *e.g.* by HRMS, and, combines the equilibration step for hydrazone formation with 14-3-3*η* screening (Fig. 2a). A number of hydrazones exhibiting lower anisotropy than *h*DMX_361–374_^pSer367^ were identified as hits, and we selected two bearing handles for further future functionalization to be assessed: *h*DMX_361–369_^pSer367^Gly-hydrazone-FC45 and *h*DMX_361–369_^pSer367^Gly-hydrazone-SIG17 (Fig. 2a, orange boxes). To confirm the single-concentration results, a competition assay was performed (Fig. 2b). *h*DMX_361–369_^pSer367^Gly-hydrazide was equilibrated with 5 equivalents of each aldehyde (product formation confirmed by LC-MS, see Fig. S4, ESI†), and serially diluted against the tracer peptide (*h*DMX_361–374_^pSer367^*) and 14-3-3*η* protein. The IC_50_ values for *h*DMX_361–369_^pSer367^Gly-hydrazone-FC45 (IC_50_ = 10.4 ± 0.3 μM) and *h*DMX_361–369_^pSer367^Gly-hydrazone-SIG17 (IC_50_ = 13.7 ± 0.5 μM) were found to be similar to *h*DMX_361–374_^pSer367^ (IC_50_ = 8.5 ± 0.2 μM) and *h*DMX_361–369_^pSer367^Gly-hydrazide (IC_50_ = 8.2 ± 1.3 μM). These data suggest the small-molecule fragments are tolerated adjacent to the peptide sequence, but that they likely contribute little to the binding energy possibly due to minimal contribution of the residues they replaced in the template peptide sequence.

**Fig. 2:**
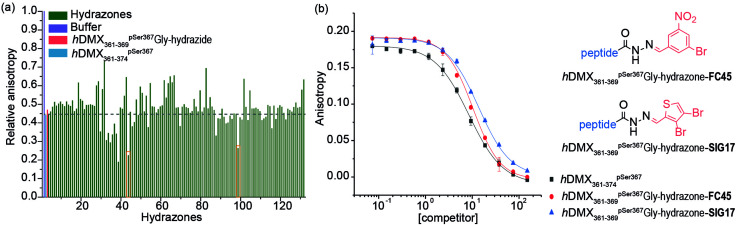
Dynamic ligation screening based on *h*DMX_361–369_^pSer367^Gly-hydrazide (a) Anisotropy values of individually screened hydrazones (relative to buffer, zero activity negative control in grey), *h*DMX_361–374_^pSer367^ (blue) and *h*DMX_361–369_^pSer367^Gly-hydrazide (red) as positive controls with hits (*h*DMX_361–369_^pSer367^Gly-hydrazone-FC45 and *h*DMX_361–369_^pSer367^Gly-hydrazone-SIG17) highlighted in orange boxes (10 μM acetylated hydrazide peptide mixed with 5 eq. of aldehyde, 1 μM 14-3-3*η*, 50 nM *h*DMX_361–374_^pSer367^*, 50 mM NH_4_OAc, 10 mM aniline and 1 mM DTT) (b) competition FA curves for hydrazones taken forward as hits (50 nM *h*DMX_361–374_^pSer367^*, 1 μM 14-3-3*η* in 50 mM NH_4_OAc, 10 mM aniline and 1 mM DTT).

To demonstrate the approach could be used to target specific pockets on a protein, the Fusicoccin pocket on 14-3-3 was then targeted with an extended library of aldehydes (165 in total). Two longer hydrazide peptides were synthesized (Fig. 1c) extended by one (*h*DMX_361–370_^pSer367^Gly-hydrazide) and two (*h*DMX_361–371_^pSer367^Gly-hydrazide) residues so as to reach the Fusicoccin pocket. Similar results (in terms of potency) were obtained for each peptide in the dynamic ligation screen; thus for clarity, only data for *h*DMX_361–370_^pSer367^Gly-hydrazide are described here (Fig. 3a; screening and validation, including for *h*DMX_361–371_^pSer367^Gly-hydrazide is shown in the ESI† Fig. S5 and S6). More hits with a *h*DMX_361–374_^pSer367^*/14-3-3*η* response greater than the control were identified in this second round. Different fragments were identified as hits for each sequence pointing to the specificity of the selection process. For validation, a competition assay for ten hits was carried out (highlighted in the orange box in Fig. 3a, ESI† for additional results, Fig. S5). Fig. 3b illustrates IC_50_ data for four hydrazone hits that exceeded affinities of *h*DMX_361–374_^pSer367^* and the control hydrazide peptide (note: the steep Hill slope for the first derivative may suggest the boronic acid plays a role in covalent modification).

**Fig. 3:**
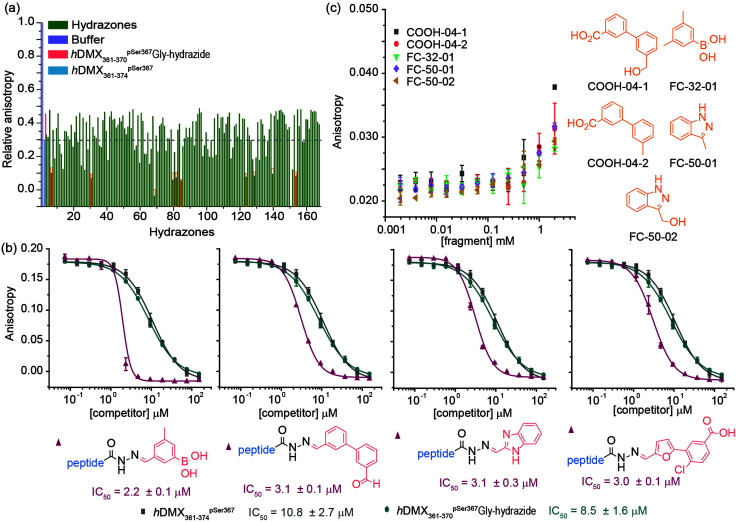
Dynamic ligation screening based on *h*DMX_361–370_^pSer367^Gly-hydrazide (a) anisotropy values of individually screened hydrazones (relative to buffer, zero activity negative control in grey), *h*DMX_361–374_^pSer367^ (blue) and *h*DMX_361–370_^pSer367^Gly-hydrazide (red) as positive controls with hits highlighted in black boxes (10 μM acetylated hydrazide peptide mixed with 5 eq. of aldehyde, 1 μM 14-3-3*η*, 50 nM *h*DMX_361–374_^pSer367^*, 50 mM NH_4_OAc, 10 mM aniline and 1 mM DTT) (b) representative competition FA curves for hydrazones taken forwards as hits (50 nM *h*DMX_361–374_^pSer367^*, 1 μM 14-3-3*η* in 50 mM NH_4_OAc, 10 mM aniline and 1 mM DTT). (c) Reduced fragments resembling hit aldehydes were chosen assess if a stabilization affect could be observed using FA (0.1 μM 14-3-3*η*, 50 nM *h*DMX_361–370_^pSer367^Gly* in 10 mM HEPES, 150 mM NaCl, 0.1% Tween 20, 0.1% BSA, pH 7.4).

To explore the effect of non-linked fragments, 5 commercially available fragments with similarity to the reduced aldehyde form of COOH4, FC50 and FC32; analogues bearing alcohol or methyl groups in place of the aldehyde (Fig. 3c) were assessed. For consistency a modified tracer peptide was used (*h*DMX_361–370_^pSer367^Gly* *i.e.* analogous to the screening peptide but bearing FAM-Ahx). The 14-3-3 affinity of this peptide was determined by FA (*K*_d_ = 172.2 ± 9.3 nM, Fig. S7, ESI†), and activity of fragments tested in serial dilution. Stabilization of the peptide/14-3-3*η* interaction was observed as the anisotropy increased at the highest fragment concentration, consistent with millimolar affinities (Fig. 3c).

## Conclusions

We used a ligand-directed dynamic-ligation approach to identify weak binding fragments that can augment the inhibitory potency of a peptide ligand and simultaneously serve as starting points for subsequent development of PPI stabilizers using the *h*DMX_361–371_^pSer367^/14-3-3*η* interaction as a model. We used acylhydrazone exchange, involving acyl hydrazide peptides and readily available aldehydes together with the surface of 14-3-3*η* protein to template peptide-fragment hybrids. Screening of representative fragments validated the method as a conceptually distinct approach for identification of PPI stabilizers. Although here, the screen was directed towards a known stabilizer pocket on 14-3-3, we note that inhibitory peptide-fragment hybrids could also reveal fragments that inhibit when separated from the peptide anchor. The acylhydrazide peptides described in this work varied in length and were designed to target distinct pockets on the 14-3-3 amphipathic groove. In obtaining different fragments from these screens, we demonstrated the site-selective specificity of the method. Future studies will centre on elaborating the identified fragments as small molecule stabilizers and broadening the approach to other targets.

## Author contributions

S. S., S. L. W. and A. J. W. conceived and designed the research program, S. S. designed studies and performed the research. The manuscript was written by S. S. and A. J. W. and edited into its final form by S. L. W. and A. J. W. with contributions from all authors.

## Conflicts of interest

There are no conflicts to declare.

## Supplementary Material

CB-003-D2CB00025C-s001
