# Supplementary material for: Towards identification of protein–protein interaction stabilizers via inhibitory peptide-fragment hybrids using templated fragment ligation
Source: RSC Chem Biol. 2022 Apr 1;3(5):546–50. doi: 10.1039/d2cb00025c (PMC9092428; doi:10.1039/d2cb00025c)
Supplement: CB-003-D2CB00025C-s001 [file CB-003-D2CB00025C-s001.pdf]

Electronic Supplementary Material (ESI) for RSC Chemical Biology.
This journal is © The Royal Society of Chemistry 2022

Towards Identification of Protein-Protein Interaction Stabilizers Using Ligand-Directed Fragment Ligation

Sonja Srdanovic,[a,b] Zsofia Hegedüs,[a,b,c] Stuart L. Warriner,[a,b] Andrew J. Wilson,[a,b*]

[a]Astbury Centre for Structural Molecular Biology, University of Leeds, Woodhouse Lane, Leeds LS2 9JT, UK

[b]School of Chemistry, University of Leeds, Woodhouse Lane, Leeds LS2 9JT, UK

[c]Department of Medical Chemistry, University of Szeged, Dóm tér 8, H-6720 Szeged, Hungary

*e-mail: a.j.wilson@leeds.ac.uk,

Supporting Information

Contents

Additional Figures 2
Experimental Methods 7
  Peptide synthesis 7
  Protein expression and purification 9
  Fluorescence anisotropy 9
  Dynamic Ligation Screen and Data Analyses 10
Peptide analytical characterization 11
References 12

Additional Figures

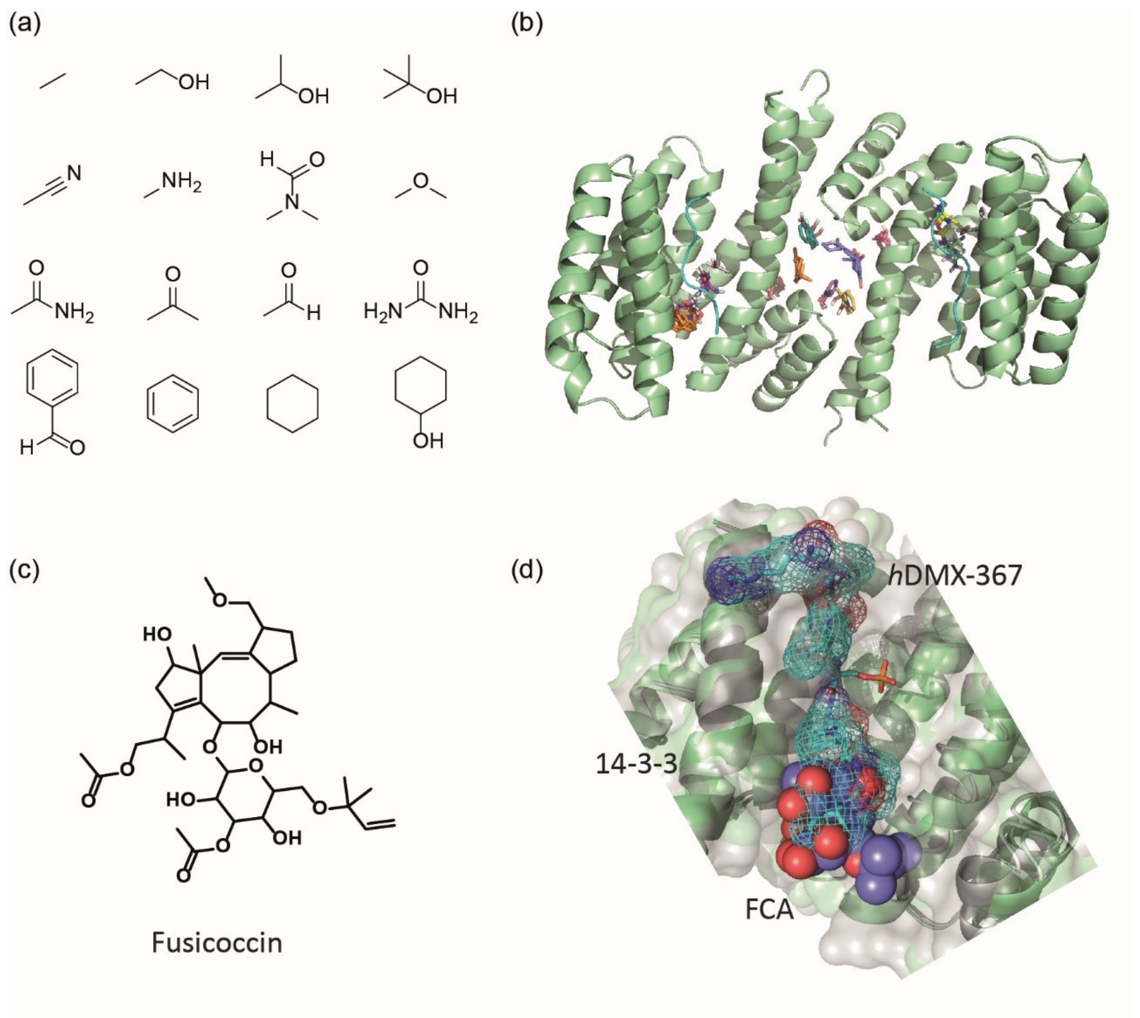

Figure S1 FTMap solvent mapping on *h*DMX$_{361\text{-}374}^{pSer367}$/14-3-3σ-ΔC co-crystal structure and comparison to Fusicoccin binding site. (a) Solvent probe molecules; (b) results obtained using FTMap; only one interesting pocket between protein and peptide was observed with a cluster of probes (protein shown in green, peptide in cyan), (c) structure of Fusicoccin (d) Overlay of *h*DMX$_{361\text{-}374}^{pSer367}$/14-3-3σ structure (PDB: 6YR5) and a Fusicoccin/14-3-3σ structure (PDB ID: 3IQV), Protein is shown in green, peptide in cyan and FCA in purple

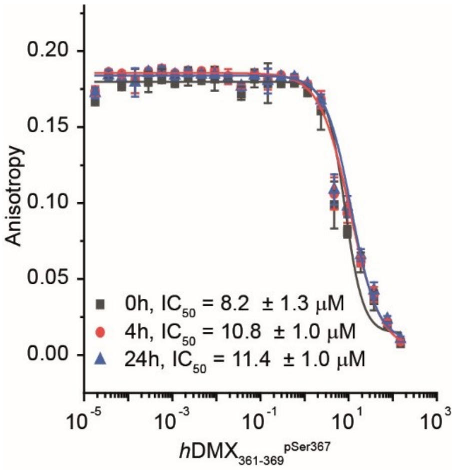

Figure S2. Competition assay for *h*DMX$_{361\text{-}369}$^{pSer367}Gly-hydrazide (ammonium acetate buffer (50 nM *h*DMX$_{361\text{-}374}$^{pSer367}*, 1 μM 14-3-3η in 50 mM NH4OAc, 10 mM aniline and 1 mM DTT)

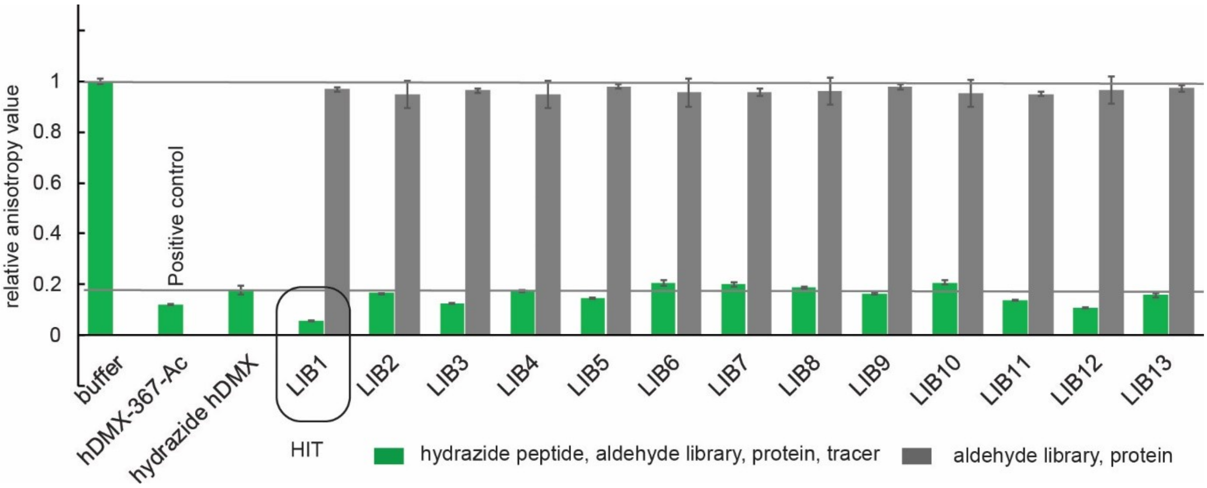

Figure S3. Preliminary dynamic ligation screening analyses. Aldehydes were assessed as 10-member cocktails (*h*DMX$_{361\text{-}369}$^{pSer367}Gly-hydrazide 100 μM, 5 eq. aldehydes, 50 nM *h*DMX$_{361\text{-}374}$^{pSer367}*, 1 μM 14-3-3η, 50 mM NH_4OAc, 10 mM aniline and 1 mM DTT). Anisotropy is shown relative to buffer control, with hydrazone libraries and control aldehydes are compared to *h*DMX$_{361\text{-}369}$^{pSer367}Gly-hydrazide and *h*DMX$_{361\text{-}374}$^{pSer367}*.

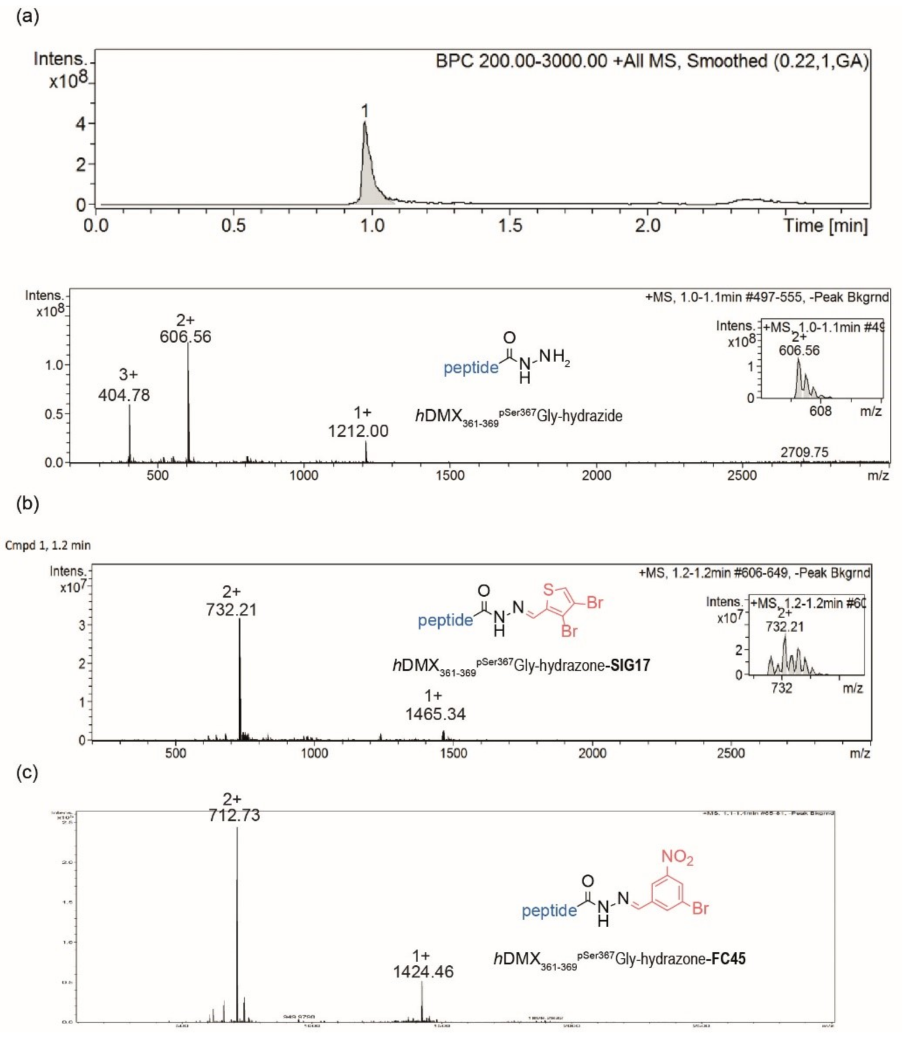

Figure S4. LC-MS analyses confirming assembly of peptide-fragment hybrids prior to dose response fluorescence anisotropy competition.

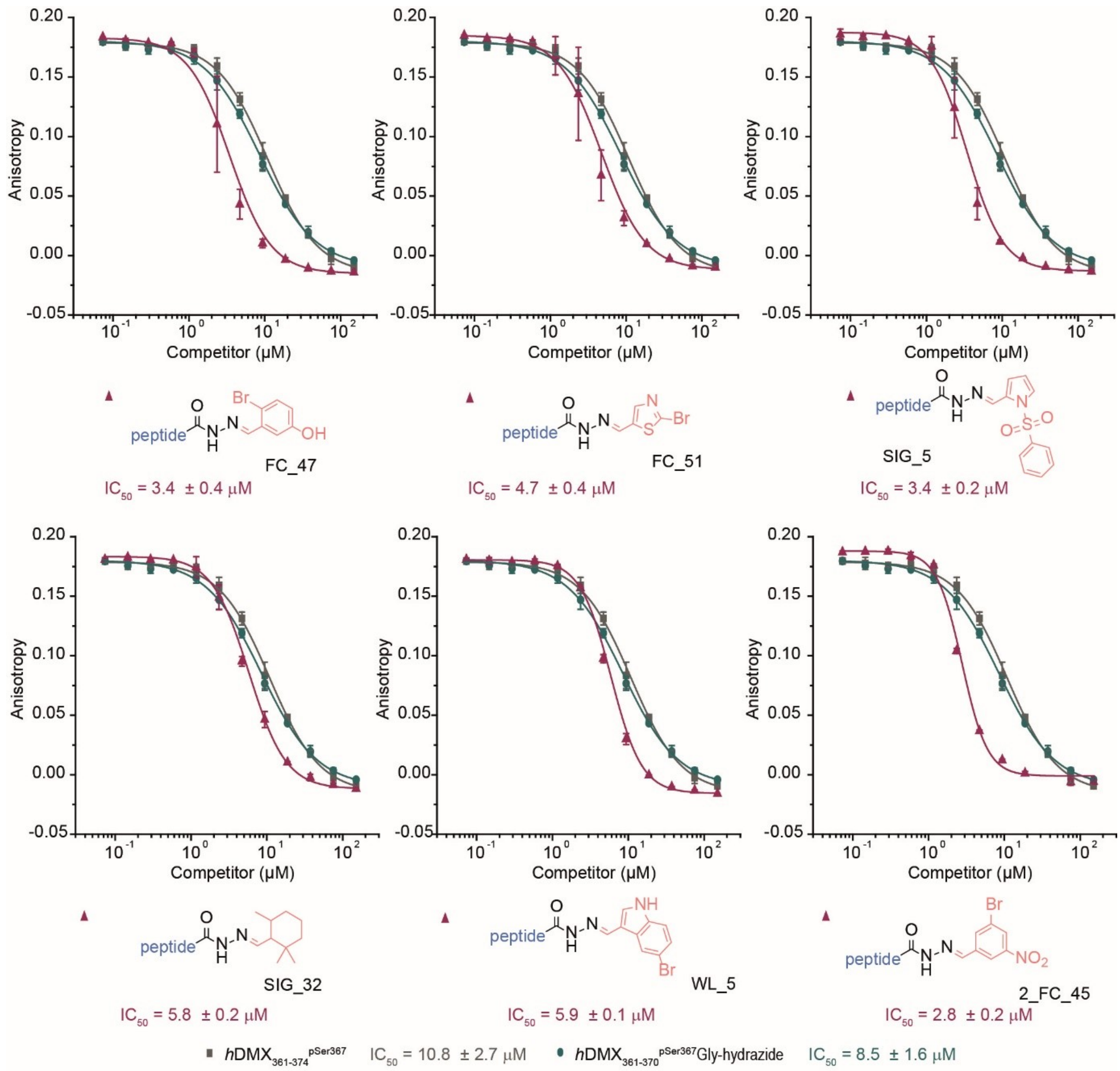

Figure S5. Dynamic ligation screening based on $h\mathrm{DMX}_{361\text{-}370}{}^{\mathrm{pSer367}}$Gly-hydrazide: additional competition FA curves for hydrazones taken forwards as hits (50 nM $h\mathrm{DMX}_{361\text{-}374}{}^{\mathrm{pSer367}*}$, 1 μM 14-3-3η in 50 mM NH_4OAc, 10 mM aniline and 1 mM DTT).

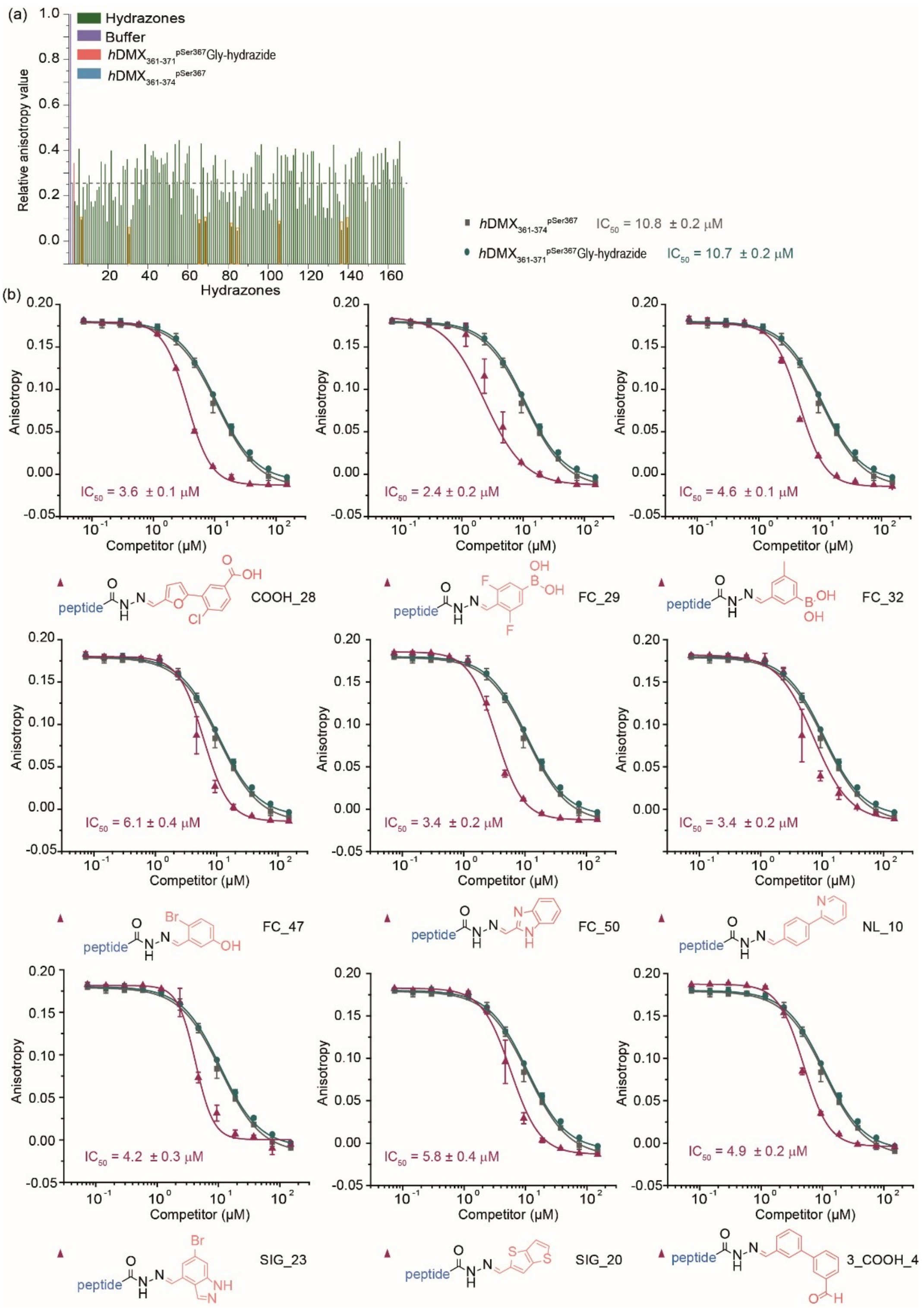

Figure S6. Dynamic ligation screening based on *h*$DMX_{361\text{-}371}{}^{pSer367}$Gly-hydrazide (a) Anisotropy values of individually screened hydrazones (relative to buffer, zero activity negative control in grey), *h*$DMX_{361\text{-}374}{}^{pSer367}$ (blue) and *h*$DMX_{361\text{-}371}{}^{pSer367}$Gly-hydrazide (red) as positive controls with hits highlighted in black boxes (10 μM acetylated hydrazide peptide mixed with 5 eq. of aldehyde, 1 μM 14-3-3η, 50 nM *h*$DMX_{361\text{-}374}{}^{pSer367*}$, 50 mM NH_4OAc, 10 mM aniline and 1 mM DTT) (b) representative competition FA curves for hydrazones taken forwards as hits (50 nM *h*$DMX_{361\text{-}374}{}^{pSer367*}$, 1 μM 14-3-3η in 50 mM NH_4OAc, 10 mM aniline and 1 mM DTT).

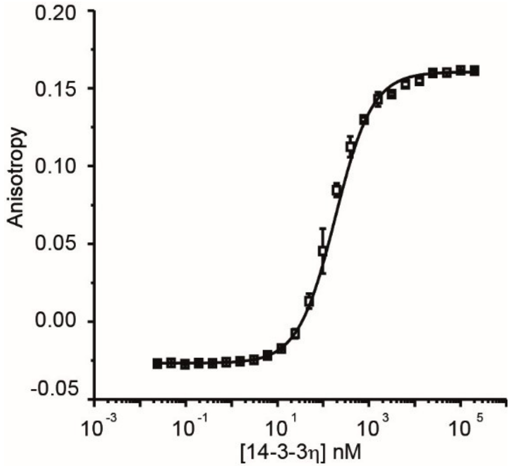

Figure S7. Direct titration of peptide for analysis of fragment stabilizing effects (*h*DMX$_{361-370}$^{pSer367}Gly* 50 nM, in 10 mM HEPES, 150 mM NaCl, 0.1% Tween 20, 0.1% BSA, pH 7.4.

Experimental Methods

Peptide synthesis

Synthesis of *h*DMX$_{361\text{-}374}^{pSer367}$ and *h*DMX$_{361\text{-}374}^{pSer367*}$ were described previously.[1]

General remarks: Resins and amino acids were purchased from either Sigma–Aldrich or Novabiochem. All amino acids were N -Fmoc protected and side chains protected with Boc (His, Lys), t-Bu (Asp, Glu, Ser, Thr), Pbf (Arg), Trt (Asn, Gln). Peptides were synthesized either manually or using a microwave assisted automated peptide synthesizer (CEM Liberty Blue) on a 0.05 or 0.1 mmol scale. DMF used in peptide synthesis was of ACS grade and from Sigma–Aldrich. C-Terminal amides were prepared using Rink Amide Resin, C-terminal acylhydrazides were prepared using hydrazone resin based on the acylation of aminomethyl polystyrene resin by the Fmoc-protected hydrazone of pyruvic acid (Fmoc-NH-N=Pyv-OH).

Automated peptide synthesis method: Peptides prepared using automated peptide synthesizer followed cycles described below. Resin loading cycle cleans the reaction vessel, washes with DMF:DCM (1:1), transfers resin to reaction vessel, washes with DMF:DCM (1:1), and drains the vessel at the end of a cycle. Deprotection and coupling cycle consist of: washing with DMF (4 ml), adding 20% piperidine in DMF (6 ml), microwave deprotection cycle (30 sec), washing with DMF (4+4+4+4 mL), addition of amino acid (2.5 ml, 5 eq or 3 eq for phosphorylated amino acids), coupling reagent (1 ml, 5 eq) and activator base (0.5 ml, 5 eq), coupling microwave cycle (5 min), washing with DMF (2 ml) and draining. HCTU and DIPEA were used during automated peptide synthesis as well. As a rule, all amino acids were coupled using 75°C coupling and deprotection cycles up to Ser(PO(OBzl)OH)-OH or Thr(PO(OBzl)OH)-OH, where conventional coupling and deprotection method was used (coupling at the rt for 2 h, deprotection at rt for 15 min) as well for every amino acid following pSer/pThr. After the final residue was coupled, the resin was ejected from the reaction vessel. Ahx coupling, deprotection, acetylation or florescent dye coupling, and cleavage was performed manually using methods described above.

Peptide purification: Crude peptides were dissolved in H_2O or DMSO and purified by UV- or MS- directed HPLC. Jupiter Proteo (250 x 21.2 mm) or a Kinetex EVO C18 (250 x 21.2 mm) preparative column (reversed phase) was used with increasing gradient of acetonitrile in water with 0.1% formic acid, over 30 min at the flowrate of 10 ml/min. Fractions containing peptide were combined, concentrated, and lyophilized. Purity of peptides was assessed by analytical HPLC and HRMS.

Manual peptide synthesis: Manual peptide synthesis followed this cycle: swelling of a resin (20 min) in cartridge used for solid-phase synthesis, washing (DMF, 3 x 2 ml x 2 min), deprotection (Method A), and coupling of a desired amino acid (Method B), where successful coupling and deprotection were determined by a colour test (Method C). Acetylation (Method D) or coupling of a fluorescent dye (Method E) were performed prior to cleavage (Method F).

Method A: Deprotection N-terminal Fmoc-protecting groups were removed by adding 20% piperidine in DMF (5 × 2 mL × 2 min) and washed with DMF (5 × 2 mL × 2 min) after.

Method B: Manual coupling of amino acid and Ahx

The desired amino acid or Ahx (5 equiv.), DIPEA (10 equiv.) and HCTU (5 equiv.) were dissolved in DMF (2 mL) and added to the resin, followed by agitation for 1 h. Reagents were removed by filtration and the resin was washed with DMF (3 × 2 mL × 2 min).

Method C: Kaiser test

Successful coupling or deprotection for any residue coupled manually was determined by Kaiser test. A few resin beads were transferred into a vial and mixed with 2 drops of each of the solutions:

1) Ninhydrin (5% w/v) in ethanol

2) Phenol (80% w/v) in ethanol

3) 1 mM KCN (aq.) in pyridine (2% v/v)

The solution was heated at 100 °C for five minutes before observing the change in colour. Successful deprotection was observed by colour of the beads changing into blue, where successful coupling gave no change in colour.

Method D: N-terminal acetylation

Acetic anhydride (10 equiv.) and DIPEA (10 equiv.) were dissolved in DMF (2 mL) and the solution was transferred to the resin. After 2 h, the resin was drained and washed with DMF (3 × 2 mL × 2 min). Successful capping was determined by colour test (Method C).

Method E: N-terminal Fluorescent Dye coupling

5,6-carboxyfluorescein (5 equiv.), DIPEA (5 equiv.) and HCTU (5 equiv.) were dissolved in DMF (2 mL) and added to the resin, followed by agitation for 1 h. Reagents were filtered and the resin was washed with DMF (3 × 2 mL × 2 min) ahead of cleavage and deprotection.

Method F: Cleavage and deprotection of peptides of the resin

After elongation and acetylation or fluorescent dye coupling was complete, the resin was washed with CH_2Cl_2 (5 × 2 mL × 2 min), Et_2O (5 × 2 mL × 2 min) and dried under vacuum.

Peptides were cleaved and side-chain deprotected using 'Reagent K' TFA:EDT:Thioanisole:Phenol:H_2O 82:3:5:5:5 (2 mL × 3 h). The peptide was precipitated in ice-cold Et_2O (10 mL) and placed in a centrifuge (3000 rpm × 5 min). The supernatant was removed, the precipitate resuspended in ice-cold Et_2O and placed in a centrifuge again (3x). The precipitate was dried under a stream of nitrogen overnight, before being dissolved in H_2O and lyophilized.

Protein expression and purification

The pProEx HTb-His-14-3-3η was expressed in BL21(DE3) cells as described previously.[1] Briefly; a single colony from a freshly made agar plate (8 g LB broth mixed with 8 g agar in 400 ml, autoclaved and poured into petri dishes to use for transformation of plasmids) was picked and mixed with 5 ml of LB media with ampicillin to inoculate a starter culture overnight at 37°C. The cells were grown in 2 L of TB media (48 g peptone, 24 g yeast, 4.6 g KH_2PO_4, 24 g $KHPO_4$, 5 ml glycerol in 2 L of dH_2O, autoclaved for 20 min at 121°C) at 37°C until the OD reached 0.4-0.6. Expression was induced by adding 0.4 mM IPTG and agitating overnight at 18°C. The expression culture was spun down (8000 rpm, 20 min, 4°C), resuspended in 200 ml of lysis buffer (50 mM Tris, 300 mM NaCl, 12.5 mM imidazole, 2 mM β-mercaptoethanol) with 5 mM $MgCl_2$ and DNAse (1:1000). The cells were lysed by French press or sonication and the solid fragments were removed by centrifugation (20000 rpm, 30 min, 4°C). The cleared lysate was loaded on a Ni^{2+}-NTA column, washed with 50 mM Tris, 300 mM NaCl, 12.5 mM imidazole, 2 mM β-mercaptoethanol, 0.1% triton X-100, and the protein was eluted with 50 mM Tris, 300 mM NaCl, 250 mM imidazole, 2 mM β-mercaptoethanol. Imidazole was removed by overnight dialysis using the Tris buffer (50 mM Tris, 300 mM NaCl, 2 mM β-mercaptoethanol), full length proteins were concentrated by centrifugation, rebuffered in HEPES buffer (25 mM HEPES pH 7.5, 100 mM NaCl, 10 mM $MgCl_2$, 0.5 mM TCEF) and stored in -80°C freezer.

Fluorescence anisotropy

Direct titration assay: All assays were performed in 384 well plates (each experiment was run in triplicates) and data were collected by Perkin Elmer EnVision 2013 plate reader with excitation at 480 nm (30 nm bandwidth), polarised dichroic mirror at 505 nm and emission at 535 nm (40 nm bandwidth, S and P polarised). Experiments were carried out in HBS buffer (10 mM HEPES, 150 mM NaCl, pH 7.4, + 0.1% Tween 20 + 0.1% BSA, or, 50 mM NH_4OAc, 10 mM aniline and 1 mM DTT). A ½ fold dilution series was performed in the titration, with plates read after 30 min, 4 h and 20-24 h. These gave consistent data and values when fitted. Collected data were processed in Microsoft Excel using the equations bellow. Total intensity I and anisotropy r were calculated using Equations 1 and 2 for each well. For protein-tracer titrations average anisotropy was plotted against protein concentration using OriginPro and

logistic curve was fitted to give r_{min} and r_{max}. Using Equation 3 anisotropy was converted into fraction bound and multiplied by peptide concentration to be fitted in Origin using Equation 5 to obtain K_d values.

Equation 1. $I = 2PG + S$

Equation 2. $$r = \frac{S - PG}{I}$$

Equation 3. $$L_b = \frac{(r - r_{min})}{\lambda(r_{max} - r) + r - r_{min}}$$

Equation 4. $$y = r_{min} + \frac{r_{max} - r_{min}}{1 + 10^{(x - logx_0)}}$$

Equation 5. $$y = \frac{((K + X + FL) - \sqrt{((K + X + FL)^2 - 4xFL))}}{2}$$

r = anisotropy, I = total intensity, P = perpendicular intensity, S = parallel intensity, L_b = fraction ligand bound, $\lambda = I_{bound}/I_{unbound} = 1$, FL = fluorescent ligand concentration, K = K_d

Competition assay: A ½ fold dilution series of the competitor was performed in the titration then tracer peptide and 14-3-3η added with plates read after 30 min, 4 h and 20-24 h. Collected data were processed as described above. Average anisotropy was plotted against competitor concentration using OriginPro and fitted to give IC_{50} values using a logistic function in the OriginPro software.

Dynamic Ligation Screen and Data Analyses

Screening was performed using fluorescence anisotropy competition assay, in 50 mM NH_4Ac pH 6.5 buffer containing 10 mM aniline, 1 mM DTT. Each sample was prepared in duplicate. Screening was performed using 10 μM acetylated hydrazide peptide mixed with 5 eq. of aldehyde, 1 μM 14-3-3η,. Aldehyde stock solutions were prepared in DMSO at 100 mM concentration and pipetted into a 96-well plate, which then was further diluted into the assay buffer to the appropriate concentration. Once reagents had been added to the plate it was sealed and hydrazone formation allowed to proceed at room temperature for 24 hours. After 24 hours, *h*DMX tracer peptide was added to a concentration of 50 nM to the experiment wells or equal amount of buffers was added to the blanks and the plate was read immediately. All plates were prepared with the following controls: i) negative control without any competitor, ii) positive controls: hydrazide or acetylated *h*DMX sequence. Anisotropy was calculated as described above, duplicates were averaged. Anisotropies were normalized relative to the negative buffer control.

Peptide analytical characterization

Table S1. Mass spectrometry data for peptides

Peptide	$[M+H]^{1+}$ Obs[d]	$[M+H]^{1+}$ Exp[d]	$[M+2H]^{2+}$ Obs[d]	$[M+2H]^{2+}$ Exp[d]	$[M+3H]^{3+}$ Obs[d]	$[M+3H]^{3+}$ Exp[d]
*h*DMX$_{361\text{-}369}$^{pSer367}-Gly-hydrazide	1212.00	1212.54	606.56	606.78	404.78	404.85
*h*DMX$_{361\text{-}370}$^{pSer367}-Gly-hydrazide	1310.60	1310.60	655.80	655.80	N/A	437.53
FAM-Ahx-*h*DMX$_{361\text{-}370}$^{pSer367}-Gly-amide	N/A	1725.72	863.41	863.36	575.90	575.91
*h*DMX$_{361\text{-}371}$^{pSer367}-Gly-hydrazide	1409.67	1409.67	705.33	705.33	470.46	470.56

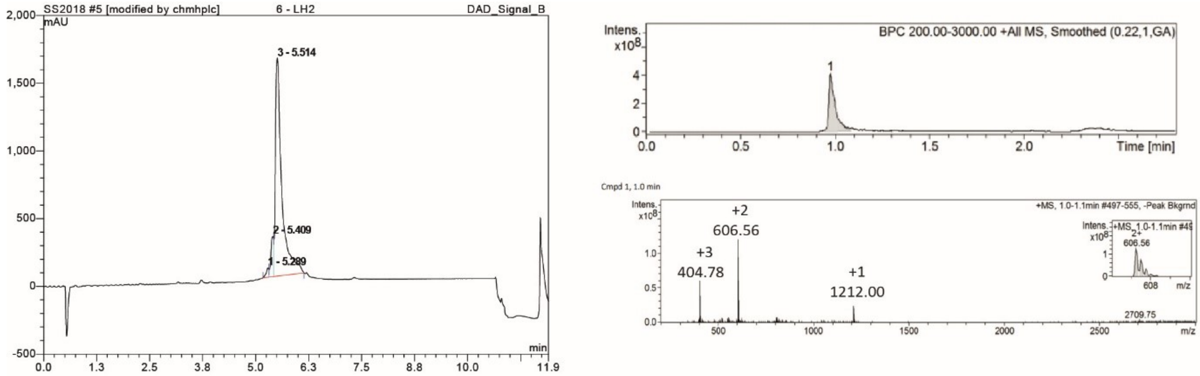

*h*DMX$_{361\text{-}369}$^{pSer367}-Gly-hydrazide

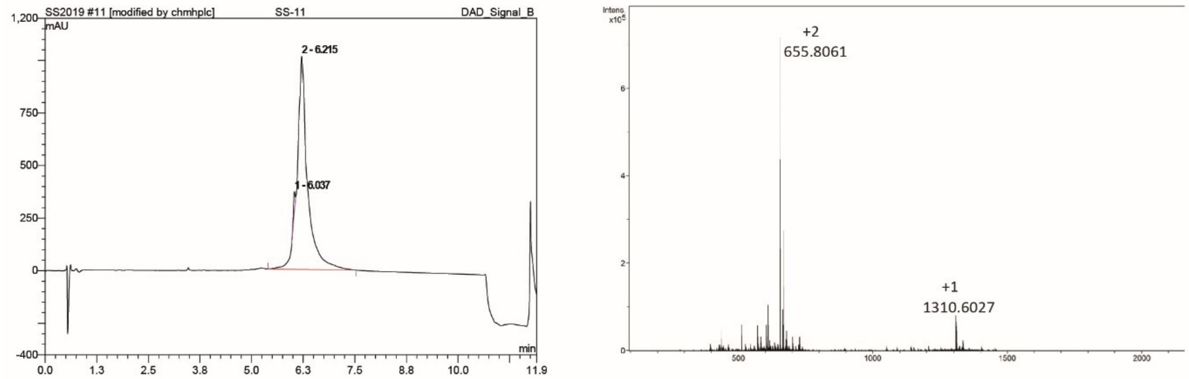

*h*DMX$_{361\text{-}370}$^{pSer367}-Gly-hydrazide

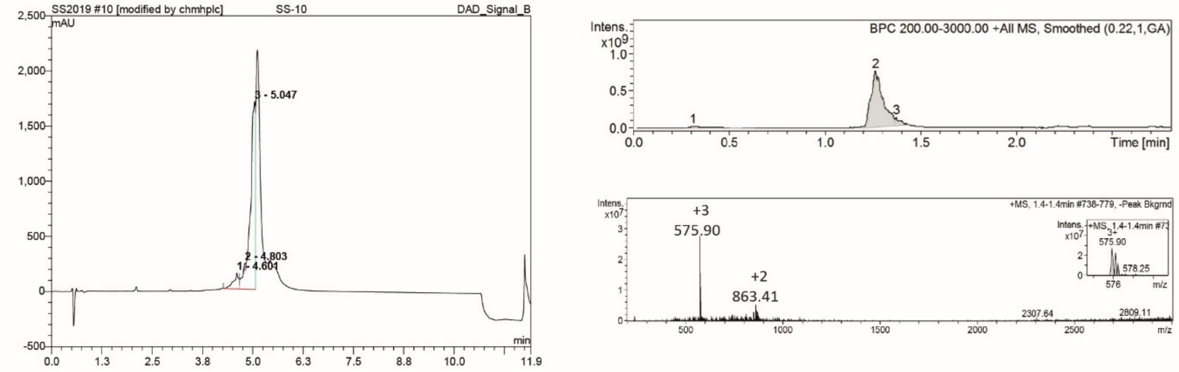

FAM-Ahx-*h*DMX$_{361\text{-}370}$^{pSer367}-Gly-amide

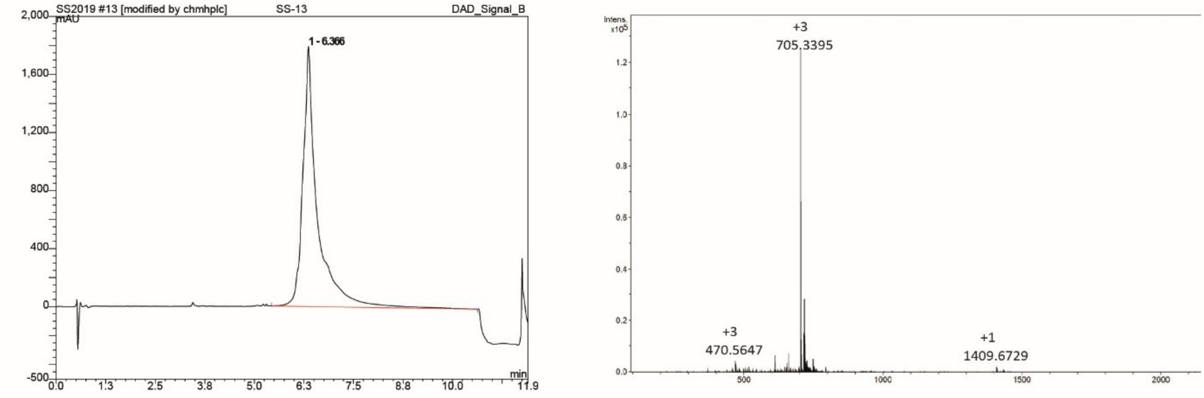

*h*DMX$_{361\text{-}371}$^{pSer367}-Gly-hydrazide

References

1. S. Srdanovic, M. Wolter, C. H. Trinh, C. Ottmann, S. L. Warriner and A. J. Wilson, *FEBS J.*, 2022, DOI: https://doi.org/10.1111/febs.16433, doi:10.1111/febs.16433.
2. S. Srdanovic, M. Wolter, C. H. Trinh, C. Ottmann, S. L. Warriner and A. J. Wilson, *bioRxiv*, 2021, DOI: 10.1101/2021.12.17.473238, 2021.2012.2017.473238.
